# Increased E-selectin in hepatic ischemia-reperfusion injury mediates liver metastasis of pancreatic cancer

**DOI:** 10.3892/or.2012.1896

**Published:** 2012-07-03

**Authors:** KATSUHIRO YOSHIMOTO, HIDEHIRO TAJIMA, TETSUO OHTA, KOICHI OKAMOTO, SEISHO SAKAI, JUN KINOSHITA, HIROYUKI FURUKAWA, ISAMU MAKINO, HIRONORI HAYASHI, KEISHI NAKAMURA, KATSUNOBU OYAMA, MASAFUMI INOKUCHI, HISATOSHI NAKAGAWARA, HIROSHI ITOH, HIDETO FUJITA, HIROYUKI TAKAMURA, ITASU NINOMIYA, HIROHISA KITAGAWA, SACHIO FUSHIDA, TAKASHI FUJIMURA, TOMOHIKO WAKAYAMA, SHOICHI ISEKI, KOICHI SHIMIZU

**Affiliations:** 1 Department of Gastroenterologic Surgery, Division of Cancer Medicine, Graduate School of Medical Science, Kanazawa University, Kanazawa; 2 Department of Histology and Embryology, Division of Cancer Medicine, Graduate School of Medical Science, Kanazawa University, Kanazawa; 3 Department of Surgery, Toyama Prefectural Central Hospital, Toyama, Japan

**Keywords:** ischemia-reperfusion injury, E-selectin, sialyl-Lewis^A^, liver metastasis, pancreatic cancer, portal clamping

## Abstract

Several recent studies have reported that selectins are produced during ischemia-reperfusion injury, and that selectin ligands play an important role in cell binding to the endothelium and in liver metastasis. Portal clamping during pancreaticoduodenectomy with vessel resection for pancreatic head cancer causes hepatic ischemia-reperfusion injury, which might promote liver metastasis. We investigated the liver colonization of pancreatic cancer cells under hepatic ischemia-reperfusion and examined the involvement of E-selectin and its ligands. A human pancreatic cancer cell line (Capan-1) was injected into the spleen of mice after hepatic ischemia-reperfusion (I/R group). In addition, to investigate the effect of an anti-E-selectin antibody on liver colonization in the IR group, mice received an intraperitoneal injection of the anti-E-selectin antibody following hepatic ischemia-reperfusion and tumor inoculation (IR+Ab group). Four weeks later, mice were sacrificed and the number of tumor nodules on the liver was compared to mice without hepatic ischemia-reperfusion (control group). The incidence of liver metastasis in the I/R group was significantly higher (16 of 20, 80%) than that in the control group (6 of 20, 30%) (P<0.01). Moreover, mice in the I/R group had significantly more tumor nodules compared to those in the control group (median, 9.9 vs. 2.7 nodules) (P<0.01). In the I/R+Ab group, only 2 of 5 (40%) mice developed liver metastases. RT-PCR and southern blotting of the liver extracts showed that the expression of IL-1 and E-selectin mRNA after hepatic ischemia-reperfusion was significantly higher than the basal levels. Hepatic ischemia-reperfusion increases liver metastases and E-selectin expression in pancreatic cancer. These results suggest that E-selectin produced due to hepatic ischemia-reperfusion is involved in liver metastasis.

## Introduction

Pancreatic cancer is a leading cause of cancer-related deaths worldwide, with a 5-year survival rate of <5% (1). For patients with localized disease, radical surgery with blood vessel resection may provide long-term benefits. However, even after curative resection, patients with pancreatic cancer face a 25–50% rate of distant metastases (2). The liver is the most frequent site of metastasis from pancreatic cancer. During hematogenous metastasis, tumor cells separate from the primary site, travel through the blood stream, marginate and adhere to the vascular endothelium, and transmigrate into extravascular sites where colonization occurs. Diapedesis of tumor cells from the circulation into secondary sites is believed to occur through a mechanism similar to that of leukocyte extravasation, in which cells must first contact and then roll along the endothelial cell layer. Rolling requires interactions between selectin cell adhesion molecules and their ligands (3,4). Selectin-mediated tumor cell adhesion has been modeled *in vitro*, with colorectal adenocarcinoma cells binding to cytokine-activated vascular endothelium (5–9). The fucosylated ligand components sialyl-Lewis^X^ (sLe^X^) and sialyl-Lewis^A^ (sLe^A^) found on the surface of circulating adenocarcinoma cells have been shown to bind to endothelial selectin (6,9–14). Studies have shown that anti-sLe^X^(10) and anti-sLe^A^(6,15) antibodies block adhesion of epithelial cancer cell lines to human umbilical vein endothelial cells (HUVEC), demonstrating the specificity of this interaction.

Hepatic ischemia-reperfusion always occurs during liver transplantation and liver resection using the Pringle method. During pancreaticoduodenectomy (PD) with portal vein resection for pancreatic cancer, portal vein clamping is used to reduce intraoperative bleeding. However, this portal clamping causes identical hepatic ischemia-reperfusion injury. Several studies have shown that hepatic ischemia-reperfusion induces free radicals and inflammatory cytokines (16–19); therefore, hepatic ischemia-reperfusion may promote liver metastasis (20).

The major objective of this study was to investigate the early molecular events in liver colonization triggered by hepatic ischemia-reperfusion that could subsequently determine the course of metastasis.

## Materials and methods

### Animals

ICR nude mice weighing 20–40 g were purchased from Charles River Japan Inc. (Kanagawa, Japan) and were maintained in our Animal Care Center. All animals were given free access to food and water. All animal experiments were performed according to Guidelines for the Care and Use of Laboratory Animals of the Kanazawa University.

### Tumor cells

The Capan-1 human pancreatic cancer cell line, which was isolated from a liver metastasis of a well-differentiated pancreatic ductal adenocarcinoma in a 40-year-old Caucasian male, was obtained from the American Type Culture Collection (Rockville, MD, USA). This cell line was maintained in media supplemented with 2 mM L-glutamine, 10% fetal calf serum (FCS), 100 U/ml penicillin and 100 μg/ml streptomycin. Cells were grown at 37°C in an atmosphere of 95% air and 5% CO_2_. Tumor cells were suspended in phosphate-buffered saline (PBS) at a density of 2×10^6^ cells/ml. Each mouse received an intrasplenic injection of 1×10^6^ cells according to a previously described method (21). Cell viability was assessed with trypan blue prior to injection.

### Treatment of animals

Mice were anesthetized with diehtylether, and the abdomen was incised to expose the liver. Total hepatic ischemia was induced by clamping the hepatic artery, portal vein, and bile duct. Ischemia was detected visually by color changes in the liver. The ischemic time was 20 min, which was well tolerated by the mice, and the reperfusion time was 15 min. The mice were divided into 2 groups: the ischemia-reperfusion (I/R) group (n=20) and the control group (no ischemia-perfusion, n=20). After hepatic ischemia-reperfusion, 1×10^6^ tumor cells in 0.5 ml of PBS were injected into the spleen of the mice. The mice were then closed with a 4.0 silk suture in a continuous running fashion. In the control group, the tumor cells were injected immediately after laparotomy without hepatic ischemia-reperfusion. The animals were sacrificed 4 weeks later. The liver was removed and the number of tumor nodules on the liver surface was counted.

In addition, to test the effect of anti-E-selectin antibody on liver colonization, another 5 animals were inoculated with 3 intraperitoneal injections of 100 μg of affinity-purified anti-E-selectin antibody at 0, 3 and 6 h after hepatic ischemia-reperfusion and tumor inoculation. The number of tumor nodules was then counted (I/R+Ab group, n=5).

To determine whether hepatic ischemia-reperfusion caused changes in local cytokine production and E-selectin mRNA expression in the liver, hepatic ischemia-reperfusion was induced, and livers were removed at different time intervals (0, 0.25, 0.5, 1, 3, 6, 9, 12 and 24 h) after ischemia-reperfusion. IL-1, TNF-α and E-selectin mRNA levels were analyzed by reverse transcription-polymerase chain reaction (RT-PCR) and southern blotting. Liver fragments were frozen immediately in liquid nitrogen and stored at −80°C until RNA extraction.

### Oligonucleotide primers and probes

The sequences of the oligonucleotides used for RT-PCR, and southern blotting were as follows. IL-1 forward, 5′-CAGATTCACAACTGTTCG TGAGCG-3′; IL-1 and reverse primer, 5′-AAGTCTGTCA TAGAG GGCAGTCCC-3′ (product size, 232 bp); IL-1 probe, 5′-CACATCAGCTGCTTATCCAGAGCTG-3′ (22); TNF-α forward, 5′-GCAGGTCTACTTTGGAGTCATTGC-3′ and reverse primer, 5′-TCCCTTTGCAGAACTCAGGAATGG-3′ (product size, 323 bp); TNF-α probe, 5′-TGTGCTCAGAGC TTTCAACAACTAC-3′ (23); E-selectin forward, 5′-GTGCG GTGTACGTCCCTCTGGAGAAGTG-3′; and reverse primer, 5′-TCCCTTTGCAGAACTCAGGAATGG-3′ (product size, 535 bp); E-selectin probe, 5′-TCAGAATCTACAGTGTACCTCATCTG-3′; GAPDH forward, 5′-GGTGAAGGTCGGTGTGAACGGATTT-3′ and reverse primer, 5′-AATGCCAAAGTTGTCATGGATGACC-3′ (product size, 502 bp); and GAPDH probe, 5′-GTGCTGAGTATGTCGTGGAGTCTAC-3′ (24).

### RT-PCR southern blot analysis

Total cellular RNA was extracted from each frozen liver fragment by the acid guanidinium thiocyanate-phenol-chloroform method (25). The concentration of total RNA was determined by measuring the OD_260_. The purity of the RNA was assessed by determining the OD_260/280_ ratio, and all samples were >1.8. Aliquots of each total RNA sample (1 μg) were subjected to reverse transcription (RT) at 42°C for 60 min using Moloney murine leukemia virus reverse transcriptase (Toyobo Inc., Osaka, Japan). Subsequently, an aliquot of each RT product was amplified in a DNA thermal cycler (MJ Research, Inc., Watertown, MA, USA), using Taq DNA polymerase (Takara Bio Inc., Shiga, Japan) and a pair of oligonucleotide primers in a final volume of 100 μl. Each amplification cycle consisted of 94°C for 1 min, 58°C for 2 min, and 72°C for 1 min and 30 sec. After 25 cycles of amplification, each RT-PCR mixture was electrophoresed on a 1.5% agarose gel and blotted onto a nylon membrane (Pall BioSupport, East Hills, NY, USA) according to the southern blot procedure (26).

An antisense oligonucleotide probe was labeled at the 3′ end with fluorescein-11-dUTP using terminal transferase (Roche Diagnostics, Basel, Switzerland) and was hybridized to the membrane at 43°C for 2 h in a shaking water bath according to the ECL 3′-oligolabelling and detection system protocol (Amersham Pharmacia Biotech, Uppsala, Sweden). After washing, the membrane was blocked with blocking solution at room temperature for 30 min, and then the membrane was incubated with antibody using diluted anti-fluorescein HRP conjugate solution containing 0.5% (w/v) bovine serum albumin at room temperature for 30 min. After the membrane was washed, the membrane was incubated with detection solution at room temperature for 10 min, and then covered with Saran wrap and after exposed to X-ray film (Kodak, Rochester, NY, USA).

### Immunocytochemical analysis of sLe^A^ antigen on Capan-1 cells

Capan-1 cells were suspended in RPMI-1640 medium on a Lab-Tek Chamber Slide (Nunc, Naperville, IL, USA) and incubated at 37°C overnight in an atmosphere of 95% air and 5% CO_2_. After washing with PBS, acetone and methanol were added and the chamber, was incubated at 4°C for 30 min. Capan-1 cells were attached to this slide, which was stored at −20°C until use. Immunocytochemical staining for sLe^A^ antigen was performed according to the EnVision technique (Dako, Glostrup, Denmark). The slide was incubated with normal goat serum in PBS for 30 min at room temperature, and then incubated with anti-human sLe^A^ monoclonal antibody (diluted 1:100) overnight at 4°C. After washing with PBS, the slide was incubated with biotinylated anti-mouse antibody for 120 min, followed by the application of peroxidase-labeled streptavidin. The reaction product was visualized with diaminobenzidine, and was counterstained with hematoxylin.

### Statistical analysis

The Chi-square and Mann-Whitney U tests were used to determine statistical significance. A P-value of <0.05 was considered significant.

## Results

### Liver colonization by Capan-1 cells after hepatic ischemia-reperfusion

**Figure 1:**
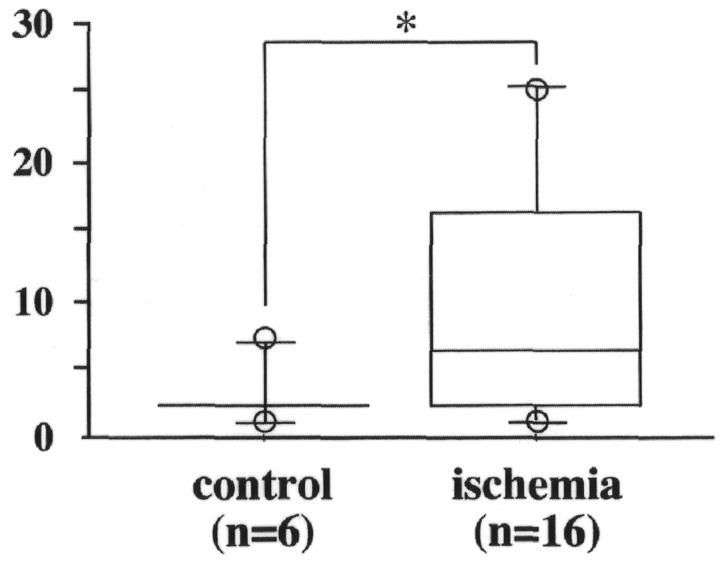
The number of liver metastases in mice with hepatic ischemia-reperfusion injury and control mice. Hepatic ischemia-reperfusion injury was induced in mice (I/R group), and then pancreatic cancer (Capan-1) cells were injected into the spleen. Control mice were injected with Capan-1 cells following laparotomy without ischemia-reperfusion. In the I/R group, more metastases per mouse were observed than in the control group.

All animals in both groups survived until sacrifice. As shown in Table I, 16 of the 20 (80%) mice in the I/R group developed hepatic metastases, which was significantly higher than the incidence of hepatic metastases in the control group (6 of 20, 30%) (P<0.01). Moreover, mice in the I/R group had more tumor nodules than in the control group (P=0.06). The number of metastases per mouse in animals positive for liver metastasis ranged from 1 to 7 (median, 2.7) in the control group, and from 1 to 25 (median, 9.9) in the I/R groups (Fig. 1).

### Effect of anti-E-selectin antibody on liver colonization after hepatic ischemia-reperfusion

As shown in Table I, injection of anti-E-selectin antibody reduced hepatic metastasis of Capan-1 cells. Only 2 of the 5 (40%) mice injected with anti-E-selectin antibody developed liver metastases and the metastases in the positive animals were 6 and 9, respectively.

### Expression of IL-1, TNF-α and E-selectin m-RNA after hepatic ischemia-reperfusion

**Figure 2:**
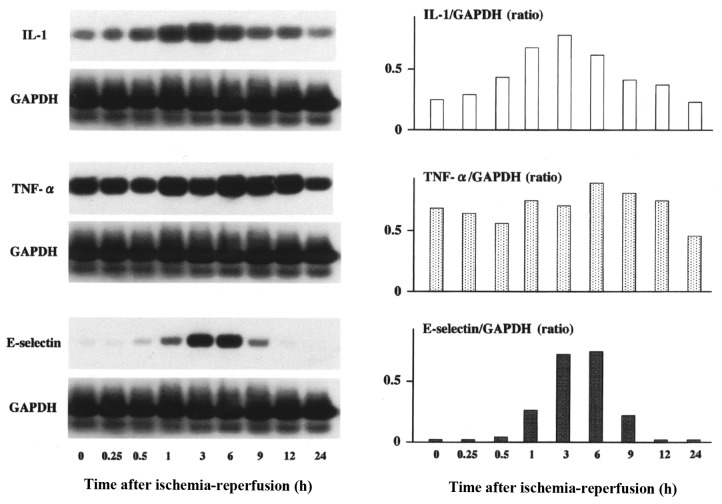
Expression of IL-1, TNF-α and E-selectin mRNA after hepatic ischemia-reperfusion. IL-1, TNF-α, and E-selectin mRNA was analyzed in liver section extracts at the indicated time points following hepatic ischemia-reperfusion using RT-PCR and southern blotting. IL-1 and E-selectin mRNAs were increased after hepatic ischemia-reperfusion. However, the expression of TNF-α mRNA was not influenced by hepatic ischemia-reperfusion.

IL-1 mRNA began to increase 30 min after ischemia-reperfusion, reached maximal levels 3 h after ischemia-reperfusion and decreased to basal levels 24 h after ischemia-reperfusion (Fig. 2). E-selectin mRNA levels began to rise within 1 h after ischemia-reperfusion, reached a maximum at 6 h, and then returned to basal levels by 24 h after hepatic ischemia-reperfusion. Hepatic ischemia-reperfusion had no significant effect on TNF-α expression at any of the time intervals examined (Fig. 2).

### Expression of aLe^A^ antigen on Capan-1 cells

**Figure 3:**
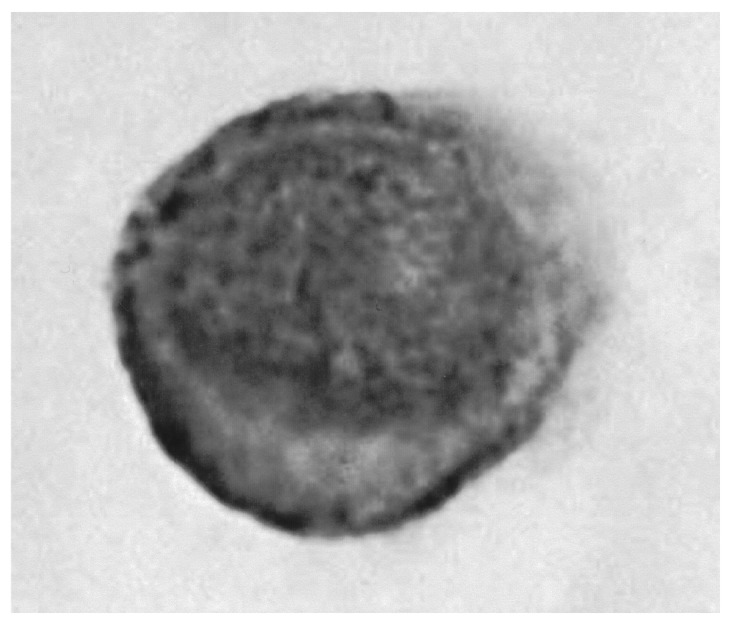
Immunocytochemical staining for siayl-Lewis^A^ antigen (sLe^A^) in the Capan-1 pancreatic cancer cell line. SLe^A^ antigen was expressed on the cell surface of Capan-1 cells.

As shown in Fig. 3, sLe^A^ antigen was expressed on the surface of Capan-1 cells.

## Discussion

Tumor metastasis is a multistep process requiring detachment of malignant cells from the primary tumor mass, penetration of blood vessels, evasion of immune surveillance, attachment to the endothelium of distant organs, penetration of the secondary tissue, and formation of new tumor colonies. The ability of disseminated cancer cells to establish metastases in secondary organs is regulated by a combination of factors including access to the organ microvasculature and specific secondary tissue-tumor cell interactions (27,28). Interaction between tumor cells and the secondary tissue endothelium is believed to be a key step in the metastatic cascade (29), and is thought to be mediated by adhesion receptor-ligand pairs, some of which are involved in physiological leukocyte-endothelial interactions (30). The various combinations of cell surface molecules expressed by tumor cells may serve as ligands for endothelial cell surface receptors (5,10,11,31,32), which are typically induced by mediators of inflammation (5,31,32). Therefore, a local inflammatory response may facilitate circulating tumor cell adhesion and arrest. Takada *et al*(9) showed that epithelial cancer cells have the ability to adhere to endothelial cells, and that their adhesion is enhanced by activation of endothelial cells with cytokines such as IL-1. They also showed that E-selectin (endothelial leukocyte adhesion molecule −1: ELAM-1), first introduced as an adhesion molecule that mediates leukocyte adhesion to endothelial cells (33), is of particular importance in the adhesion of human epithelial cancer cells to vascular endothelial cells.

E-selectin is an adhesion molecule that is not expressed on normal endothelial cells. However, E-selectin is transiently expressed on the surface of vascular endothelium after stimulation with IL-1 and TNF-α, and has been implicated in the initial events of neutrophil extravasation in the inflammatory response. E-selectin also appears to be involved in tumor invasion and metastasis, since E-selectin mediates adherence of leukemia and colon cancer cells to activated endothelial cells. Several studies have implicated E-selectin in the adhesion of cancer cells to vascular endothelial cells (6,8,10,34,35). It has been suggested that E-selectin is involved in the preferential homing of metastasizing cells to the liver (36), and that E-selectin mediates the metastasis of certain tumor types (36,37). Uotani *et al* reported that induction of E-selectin after partial hepatectomy promotes liver metastasis in mice (38).

We examined the expression of cytokines, such as IL-1 and TNF-α, and E-selectin by RT-PCR and southern blotting after hepatic ischemia-reperfusion. Our results showed maximum expression of E-selectin mRNA 6 h after hepatic ischemia-reperfusion, with a return to baseline levels by 24 h after ischemia-reperfusion. This finding is consistent with previous reports (38,39).

In liver surgery for pancreatic cancer with portal resection, temporary hepatic pedicle clamping has been used to reduce intraoperative bleeding since the report by Pringle (40). However, this clamping causes ischemia-reperfusion injury (19,41), and ischemia-reperfusion injury elicits endothelial cell injury that can manifest as swelling, detachment from the underlying basement membrane, and compromised barrier function. These events might be accompanied by leukocyte-endothelial cell adhesion, which manifest as rolling, firm adhesion, and emigration of leukocytes in post-capillary venules of the microvasculature (42). Several studies have reported that hepatic ischemic-reperfusion induces free radicals and inflammatory cytokines (16–19). Free radicals and cytokines, such as TNF-α and IL-1 are implicated in the acceleration of hepatic metastasis, and therefore it is possible that hepatic ischemia-reperfusion promotes liver metastasis (20). Our study verified that hepatic ischemia-reperfusion promotes liver metastasis of Capan-1 cells; the number of tumor nodules in the I/R group was significantly greater than that in the control group. We also found that administration of an anti-E-selectin monoclonal antibody to mice in the I/R group after tumor inoculation inhibited liver metastasis. This finding is supported by previous reports which demonstrated that a neutralizing murine E-selectin monoclonal antibody abrogated hepatic metastasis *in vivo*(36,38).

The sialylated, fucosylated tetrasaccharides Le^X^ and sLe^A^ and related carbohydrate structures, have been identified as selectin ligands (6,9–14,34). Interestingly, sLe^X^ and sLe^A^ have also been identified as markers of progression in several types of carcinoma, particularly carcinomas of the gastrointestinal tract, which commonly metastasize to the liver (43,44). *In vitro* adhesion studies have shown that carbohydrate determinants adhere to TNF-α-inducible E-selectin on HUVECs (6–9,13,36).

Our study showed that sLe^A^ was expressed on the surface of Capan-1 cells. It has been reported that sLe^A^ on pancreatic carcinoma cells is an important ligand for E-selectin on activated endothelial cells (15,45). These results suggested that sLe^A^ expression on pancreatic carcinoma cell lines might modulate events related to carcinoma cell-to-endothelial cell attachment, thereby contributing to hematogenous metastasis *in vivo*(46). A recent study reported that intermittent hepatic ischemia-reperfusion preconditioning minimizes liver metastasis (47). The usefulness of intermittent hepatic ischemia-reperfusion preconditioning in Pringle’s method for hepatectomy is well known (16–19,40). During PD with portal vein resection and reconstruction, portal vein clamping is necessary to reduce intraoperative bleeding. However, portal vein clamping-unclamping causes ischemia-reperfusion of the liver, but congestion-re-outflow in the small intestine. To avoid congestion of the small intestine, we have been using small intestinal ischemic preconditioning by clamping the superior mesenteric artery (SMA) during portal vein clamping in PD with portal vein resection. We have reported that small intestinal ischemia-reperfusion injury was reduced by ischemic preconditioning induced by clamping the SMA (48). Small intestinal ischemic preconditioning by clamping the SMA could suppress the increase in cytokines and reduce undesirable liver metastasis from pancreatic cancer due to surgical procedures such as intraoperative portal vein clamping.

In conclusion, hepatic ischemia-reperfusion causes increased liver metastases as well as increased expression of E-selectin, which has been reported to mediate metastasis of cancer cells that express sLe^A^. Agents that interfere with hepatic E-selectin induction and/or function might have therapeutic, anti-metastatic effects during the early stages of liver colonization.

## Figures and Tables

**Table I:** Incidence of liver metastasis in control mice and in mice with hepatic ischemia-reperfusion injury treated or not treated with the anti-E-selectin antibody.

	I/R group (n=20)	Control group (n=5)	I/R+Ab group (n=20)
Liver metastasis (+)	16	6	2
Incidence	80%	30%	40%

P<0.01, control group vs. the I/R group.
